# Outcome Differences of Remnant- Preserving versus Non-Preserving Methods in Arthroscopic Anterior Cruciate Ligament Reconstruction: A Meta-analysis with Subgroup analysis

**DOI:** 10.1186/s43019-019-0017-z

**Published:** 2020-01-01

**Authors:** Sung Hun Won, Byung-Il Lee, Su Yeon Park, Kyung-Dae Min, Jun-Bum Kim, Sai-Won Kwon, Yong-Beom Kim, Gi-Won Seo, Jae-Hyung Kim, Hyung-Suk Choi

**Affiliations:** 1 Department of Orthopaedic Surgery, Soonchunhyang University Hospital Seoul, 59, Daesagwan-ro, Yongsan-gu, Seoul, 04401 Korea; 2 Department of Biostatistics, Soonchunhyang University Hospital, Seoul, Korea; 3 Department of Orthopaedic Surgery, Soonchunhyang University Hospital, Bucheon, Korea; 4 Department of Orthopaedic Surgery, Soonchunhyang University Hospital, Cheonan, Korea; 5 Department of Orthopaedic Surgery, Soonchunhyang University Hospital, Gumi, Korea

**Keywords:** Anterior cruciate ligament, Reconstruction, Remnant preservation, Meta-analysis, Subgroup analysis

## Abstract

**Purpose:**

To analyze differences in clinical outcomes of arthroscopic anterior cruciate ligament reconstruction between remnant-preserving and non-preserving methods.

**Methods:**

International electronical databases PubMed, Embase, and the Cochrane central database from January 1966 to December 2017 were searched for randomized controlled trials (RCTs) and observational studies that compared differences of clinical outcomes of ACL reconstruction with and without remnant preservation. A meta-analysis of these studies was performed to compare clinical outcomes. Subgroup analyses were conducted to evaluate the role of methodological quality in primary meta-analysis estimates.

**Results:**

Five RCTs and six observational studies were included in this meta-analysis and subgroup analysis. The remnant-preserving method in arthroscopic ACL reconstruction showed a statistically significant difference compared to the non-preserving method regarding arthrometric evaluation (side-to-side difference). Lachman test, Lysholm scores, and IKDC subjective scores showed statistically minor difference in meta-analysis, but showed no significant difference in subgroup analysis. Remained parameters including pivot shift test, IKDC grades, incidence of cyclops lesion showed no statistically differences in meta-analysis or subgroup analysis.

**Conclusions:**

This meta-analysis with subgroup analysis showed that arthroscopic remnant-preserving ACL reconstruction provided statistically significant but limited clinical relevance in terms of arthrometric evaluation. Results of Lachman test, Lysholm scores, and IKDC subjective scores demonstrated statistically minor differences.

## Introduction

Anterior cruciate ligament (ACL) reconstruction is a common procedure. Surgical ACL reconstruction has greatly evolved over the last decades. Current trends are to restore the native ACL anatomy and maintain its functional ability. Preserving the ACL remnant is one of efforts toward more anatomic and biologic reconstruction.

Recently, a number of studies have suggested that remnant-preserving ACL reconstruction produces satisfactory clinical outcomes [1–3]. Theoretically, preserving ACL remnants might have advantages in terms of preserving proprioceptive mechanoreceptors, enhancing revascularization, reducing synovial fluid leakage into bone tunnels, and improving knee stability [4, 5]. These advantages can result in excellent graft reinnervation, ligamentization, remodeling, and better clinical outcomes [2, 3].

The decision of whether to perform remnant-preserving or non-preserving ACL reconstruction is dictated by unique characteristics of the ACL remnant status. Remnant-preserving ACL reconstruction requires a more comprehensive and detailed diagnostic assessment of ACL injury patterns before and during surgery with an in-depth understanding of the anatomy of ACL insertion sites. Delicate debridement and bone tunnel placement are also important for reconstructing the ACL while preserving ACL remnants. Authors of some systematic reviews [6, 7] and meta-analyses [8, 9] have reported that patients in remnant-preserving ACL reconstruction groups do not have superior results to those in non-preserving groups. However, these systematic reviews and meta-analyses only reflect results of a small number of randomized controlled trials (RCTs).

The primary outcome of this meta-analysis was to analyze published literature to compare clinical outcomes of remnant-preserving versus non-preserving ACL reconstruction methods. The secondary outcome was to perform subgroup analyses of each clinical outcome from both prospective and observational studies. The hypothesis of this study was that remnant-preserving ACL reconstruction would have better clinical outcomes than non-preserving ACL reconstruction.

## Methods

### Searching strategy

Two researchers independently searched international electronic databases PubMed, Embase, and the Cochrane central database from January 1966 to December 2017 using the following keywords: anterior cruciate ligament, ACL, remnant, preservation, and stump. Search terms used in the PubMed search are presented in Table 1. Manual searches of all references listed in identified studies were also performed.

**Table 1:** Pubmed search strategy

#1	Anterior cruciate ligament [MeSH Terms]
#2	Anterior cruciate ligament [Title/abstract]
#3	Anterior cruciate ligaments [Title/abstract]
#4	ACL [Title/abstract]
#5	#1 or #2 or #3 or #4
#6	Reconstructive surgical procedures [MeSH Terms]
#7	Reconstructive surgical procedures [Title/abstract]
#8	Reconstructive surgical procedure [Title/abstract]
#9	Reconstructive surgeries [Title/abstract]
#10	Reconstructive surgery [Title/abstract]
#11	Reconstructive operation [Title/abstract]
#12	#6 or #7 or #8 or #9 or #10 or #11
#13	Arthroscopy [MeSH Terms]
#14	Arthroscopy [Title/abstract]
#15	#13 or #14
#16	Joint instability [MeSH Terms]
#17	Joint instability [Title/abstract]
#18	#16 or #17
#19	Tendon transfer [MeSH Terms]
#20	Tendon transfer [Title/abstract]
#21	Transplantation [MeSH Terms]
#22	Transplantation [Title/abstract]
#23	Transplants [MeSH Terms]
#24	Transplants [Title/abstract]
#25	#19 or #20 or #21 or #22 or #23 or #24
#26	Grafts [Title/abstract]
#27	Augmentation [Title/abstract]
#28	Single bundle [Title/abstract]
#29	Double bundle [Title/abstract]
#30	#26 or #27 or #28 #29
#31	#5 or #12 or #15 or #18 or #25 or #30
#32	Remnant [Title/abstract]
#33	Stump [Title/abstract]
#34	Minimal debridement [Title/abstract]
#35	ACL tissue [Title/abstract]
#36	#32 or #33 or #34 or #35
#37	#31 and #36

### Selection criteria

Inclusion criteria were: [1] subjects, all adult patients who underwent arthroscopy-assisted ACL reconstruction regardless of sex or race; [2] intervention methods, arthroscopy-assisted ACL reconstruction and comparisons of clinical outcomes between remnant-preserving and non-preserving methods; [3] outcome parameters, KT-1000/2000 arthrometer (MEDmetric, San Diego, CA, USA), Rolimeter, pivot shift test, Lachman test results, Lysholm Knee Scoring Scale scores, International Knee Documentation Committee (IKDC) grades, IKDC subjective scores, cyclops lesion; and [4] study types, randomized controlled versus observational. Exclusion criteria were: [1] animal or cadaver studies; [2] comparisons not between remnant-preserving and non-preserving method in arthroscopic ACL reconstruction; and [3] studies with < 1-year of follow-up.

### Literature selection

Two researchers independently selected all articles following the above-mentioned selection criteria while assessing qualities of selected articles. Any disagreement was resolved through discussion with the corresponding researcher. The Physiotherapy Evidence Database (PEDro) scale was used for RCTs. The scale comprised 11 items based on the Delphi list to assess the methodological quality of each article. Each item was scored yes or no. Scoring system on the basis of Newcastle-Ottawa Scale (NOS) was used to assess the quality of included observational studies.

### Data extraction

Using the same format, two researchers independently extracted data from articles, compared data, and repeated extractions and comparisons for items with inconsistencies. Specifically, two researchers extracted the following information from each study: first author, year of publication, country where the study was performed, study design, number of cases and controls, age, sex, follow-up period, type of graft, number of bundles, KT-1000/2000 arthrometer measurement, Rolimeter, pivot shift test, Lachman test result, Lysholm scores, IKDC grade and subjective scores and cyclops lesion, relative risk (RRs) or standardized incidence ratio (SIRs) with 95% confidence intervals (CIs), and variables used in multivariate adjustments (Tables 2 and 3). To obtain the omitted data, we e-mailed some authors [5, 10–19]. The meta-analysis was conducted in accordance with PRISMA (Preferred Reporting Items for Systematic Review and Meta-Analysis) guidelines.

**Table 2:** Description of included trials: Demographics and operation overview

Author	Year	Country	Sample Size (P/S)	Mean Age (P/S)	Sex ratio (M/F)		Follow-up, Months (P/S)	Type of graft	No. of Bundles	Surgical technique	Journal name	Level of Evidence
					P	S						
RCT												
Gohil, et al. [10]	2007	Australia	24 / 25	36/31	14/10	13/12	12/12	autograft	SB	Transtibial	JBJS (Br)	Level I
Hong, et al. [11]	2012	China	45 / 45	34/28	33/12	34/11	26/26	allograft	SB	Transtibial	AJSM	Level I
Mohtadi, et al. [12]	2012	Canada	43 / 43	20/30	19/24	24/19	12/12	autograft	SB	Transtibial	CJSM	Level I
Pujol, et al. [13]	2012	France	29 / 25	31/29	16/13	17/8	12/12	autograft	SB	Out-side in	OTSR	Level I
										AM portal		
										Transtibial		
Zhang, et al. [5]	2014	China	27 / 24	24/25	19/4	21/5	24/25	autograft	SB	Transtibial	KSSTA	Level I
Observation study												
Qi, et al. [14]	2010	China	37 / 59	24/28	26/11	35/24	15/15	allograft	SB	Out-side in	CJRRS	Level II
Park, et al. [15]	2012	South Korea	55 / 45	30/32	45/10	40/5	34/31	autograft, allograft	SB,DB	Transtibial	Arthroscopy	Level IV
Naylor, et al. [16]	2013	Canada	45 / 45	30/30	21/24	26/19	12/12	autograft	SB	Transtibial	SOST	Level II
Takazawa, et al. [17]	2013	Japan	85 / 98	24/26	124/59		33/31	autograft	SB	Transtibial	OJSM	Level III
Chen, et al. [18]	2015	China	38 / 37	29/27	27/11	25/12	12/12	autograft	SB	Transtibial	CJTER	Level III
Kondo, et al. [19]	2015	Japan	81 / 98	29/30	44/37	54/44	14/14	autograft	DB	AM portal	AJSM	Level II

*P* Preserved group, *S* Standard group, *M* Male, *F* Female, *SB* Single bundle, *DB* Double bundle, *AM* Anteromedial, *JBJS (Br)* Journal of Bone and Joint Surgery (British), *AJSM* The American journal of sports medicine, *CJSM* Clinical Jounral of Sport Medicine, *OTSR* Orthopaedics & traumatology, surgery & research, *KSSTA* Knee Surgery, Sports Traumatology, Arthroscopy, *CJRRS* Chinese journal of reparative and reconstructive surgery, *SOST* Sport-Orthopadie – Sport-Traumatologie, *OJSM* Orthopaedic Journal of Sports Medicine, *CJTER* Chinese Journal of Tissue Engineering Research

**Table 3:** Description of included trials: Method of Evaluation

Author	Stability			Pivot shift test	Lachman test	Lysholm test	IKDC grade	IKDC subjective score	Complication	
	KT-1000	KT-2000	Rolimeter						Cyclops lesion	
									MRI	Second look arthroscopy
RCT										
Gohil, et al. [10]	O							O	O	
Hong, et al. [11]	O			O	O	O	O			O
Mohtadi, et al. [12]	O			O						
Pujol, et al. [13]			O	O	O	O	O	O		O
Zhang, et al. [5]		O				O				
Observation study										
Qi, et al. [14]		O				O		O		
Park, et al. [15]	O			O	O	O		O		
Naylor, et al. [16]		O		O	O		O	O		
Takazawa, et al. [17]		O		O						
Chen, et al. [18]		O				O		O		
Kondo, et al. [19]		O		O		O				O

*IKDC* International Knee Documentation Committee, *MRI* Magnetic resonance imaging

### Statistical methods

**Fig. 1:**
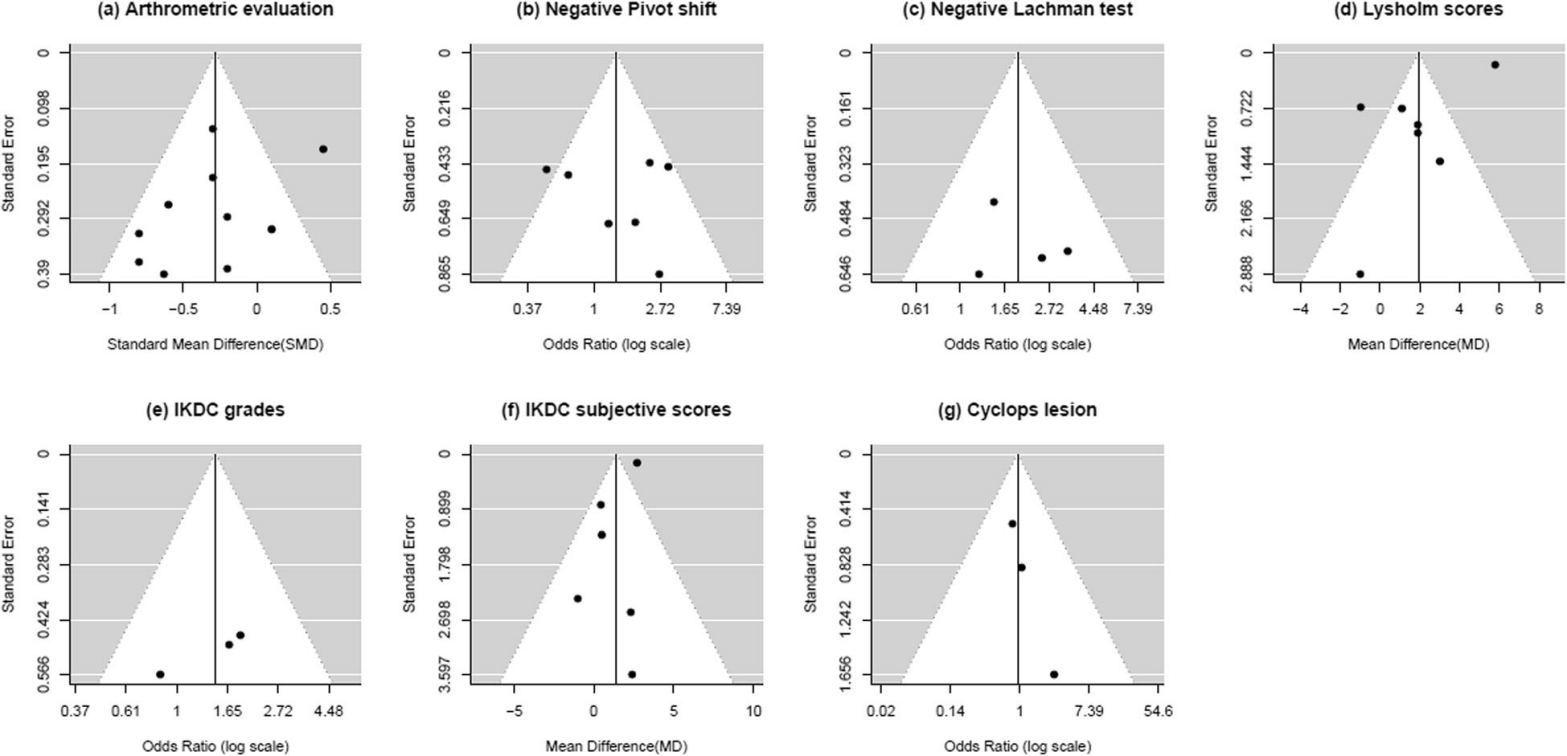
Funnel plot for publication bias

The meta-analysis was conducted using R version 3.1.2 (“metafor” and “meta” packages). For all comparisons, a random-effects model was used. Odds ratio (OR) and 95% confidence intervals (CI) were presented. OR was used to estimate the overall effect size for binary outcome. Standardized mean difference (SMD) or mean difference measure was used to calculate the overall effect size for continuous outcome. Values of SMD or MD and 95% CI were presented. Using the measure for summary statistics of continuous variable, whether the same measurements were used or not was determined. The heterogeneity of involved studies was tested with a level of significance of α = 0.10 to calculate the heterogeneity index I^2^. The estimate of I^2^ ≤ 60% was considered as low statistical heterogeneity between studies. When heterogeneity was presented, a subgroup analysis and sensitivity analysis were used to find out which study was causing the problem. Subgroup analyses were undertaken to evaluate the type of study design (RCT or observational study) in the primary meta-analysis estimate. The sensitivity analysis was performed to evaluate for the cause of heterogeneity by using results obtained when each study was excluded from all studies used in overall results. To determine the publication bias, Begg’s test and funnel plot (Fig. 1) were used. There was no evidence of significant publication bias (*P* = 0.146 for knee laxity; *P* = 0.634 for Pivot shift test; *P* = 0.649 for Lachman test; *P* = 0.203 for Lysholm scores; *P* = 0.261 for IKDC grade; *P* = 0.328 for IKDC subjective scores; and *P* = 0.480 for Cyclops lesion).

## Results

### Search results and study characteristics

**Fig. 2:**
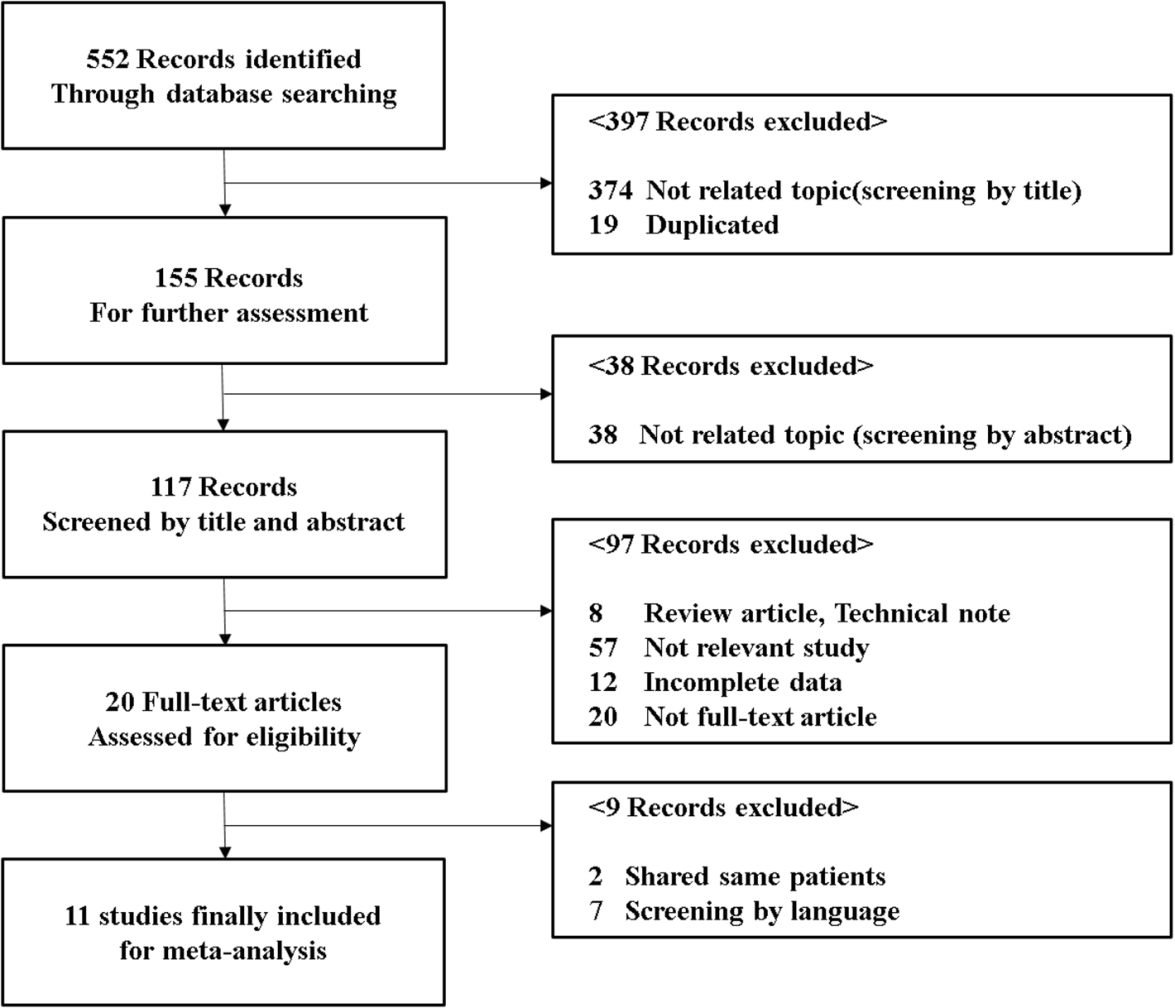
Flowchart of article selection process

Through our literature search, 552 potentially relevant studies were identified. After removing duplicate articles (19), 117 were identified by screening titles and abstracts. Finally, five RCTs [5, 10–13] and six observational studies [14–19] were included in this meta-analysis with subgroup analysis. A summary of the review process is presented in Fig. 2.

The total sample size was 1053 patients. In RCTs, 168 patients in the remnant-preserving method group and 162 patients in the non-preserving method group. In observational studies, 341 patients in the remnant-preserving method group and 382 patients in the non-preserving method group. All included studies used either autograft or allograft with different graft fixation techniques. And follow-up duration of each studies were more than 12 months. Table 2 summarized demographics and surgical details of included studies in this meta-analysis and Table 3 summarized outcome parameters of clinical evaluation of included study in this meta-analysis.

Moderate inter-rater reliability (intraclass correlation coefficients (ICC) = 0.68, 95% CI: 0.57 to 0.76%) was demonstrated by the PEDro score on RCTs. Trials with scores ≥6 were considered to be of high quality and all RCTs showed scoring of high quality (Table 4). Observational study with scores ≥7 were considered to be of high quality and all observational studies showed scoring of high quality (Table 5).

**Table 4:** PEDro critical appraisal tool results of 5 Randomized Controlled Trials

Study		PEDro Criteria											Total
		1	2	3	4	5	6	7	8	9	10	11	
RCT													
Gohil, et al. [10]	2007	N	Y	Y	Y	Y	N	Y	Y	Y	Y	Y	9
Hong, et al. [11]	2012	Y	Y	Y	Y	Y	N	N	Y	Y	Y	Y	8
Mohtadi., et al. [12]	2012	Y	Y	N	Y	Y	N	N	Y	Y	Y	Y	7
Pujol, et al. [13]	2012	Y	Y	N	Y	Y	N	N	Y	Y	Y	Y	7
Zhang, et al. [5]	2014	Y	Y	N	Y	Y	N	Y	Y	Y	Y	Y	8

*PEDro* Physiotherapy Evidence Database scale, *RCT* Randomized Controlled Trial, *Y* Yes, *N* No

Criteria: 1. eligibility criteria were specified; 2. subjects were randomly allocated to groups (in a crossover study, subjects were randomly allocated an order in which treatments were received); 3. allocation was concealed; 4. the groups were similar at baseline regarding the most important prognostic indicators; 5. there was blinding of all subjects; 6. there was blinding of all therapists who administered the therapy; 7. there was blinding of all assessors who measured at least one key outcome; 8. measures of at least one key outcome were obtained from more than 85% of the subjects initially allocated to groups; 9. all subjects for whom outcome measures were available received the treatment or control condition as allocated or, where this was not the case, data for at least one key outcome was analysed by “intention to treat”; 10. the results of between-group statistical comparisons are reported for at least one key outcome; 11. the study provides both point measures and measures of variability for at least one key outcome

**Table 5:** Newcastle-Ottawa scale for assessing the quality of 6 observational studies

		Selection			Comparability		Outcome		Total score	
	1	2	3	4	5	6	7	8		
Qi, et al. [14]	2010	★	★	★	★	★	★	★		7
Park, et al. [15]	2012	★	★	★	★	★★	★	★	★	9
Naylor, et al. [16]	2013	★	★	★	★	★★	★	★		8
Takazawa, et al. [17]	2013	★	★	★	★	★	★	★	★	8
Chen, et al [18]	2015	★	★	★	★	★	★	★		7
Kondo, et al. [19]	2015	★	★	★	★	★★	★	★		8

Criteria: 1. Representativeness of the exposed cohort; 2. Selection of the non-exposed cohort;, 3. Ascertainment of exposure; 4. Outcome not present at the start of the study; 5. Comparability; 6. Assessment of outcome, 7. Length of follow-up (study with follow-up time > 2 years was assigned one star); 8. Adequacy of follow-up (study with follow-up rate > 80% was assigned one star)

### Arthrometric evaluation (side- to- side difference)

**Fig. 3:**
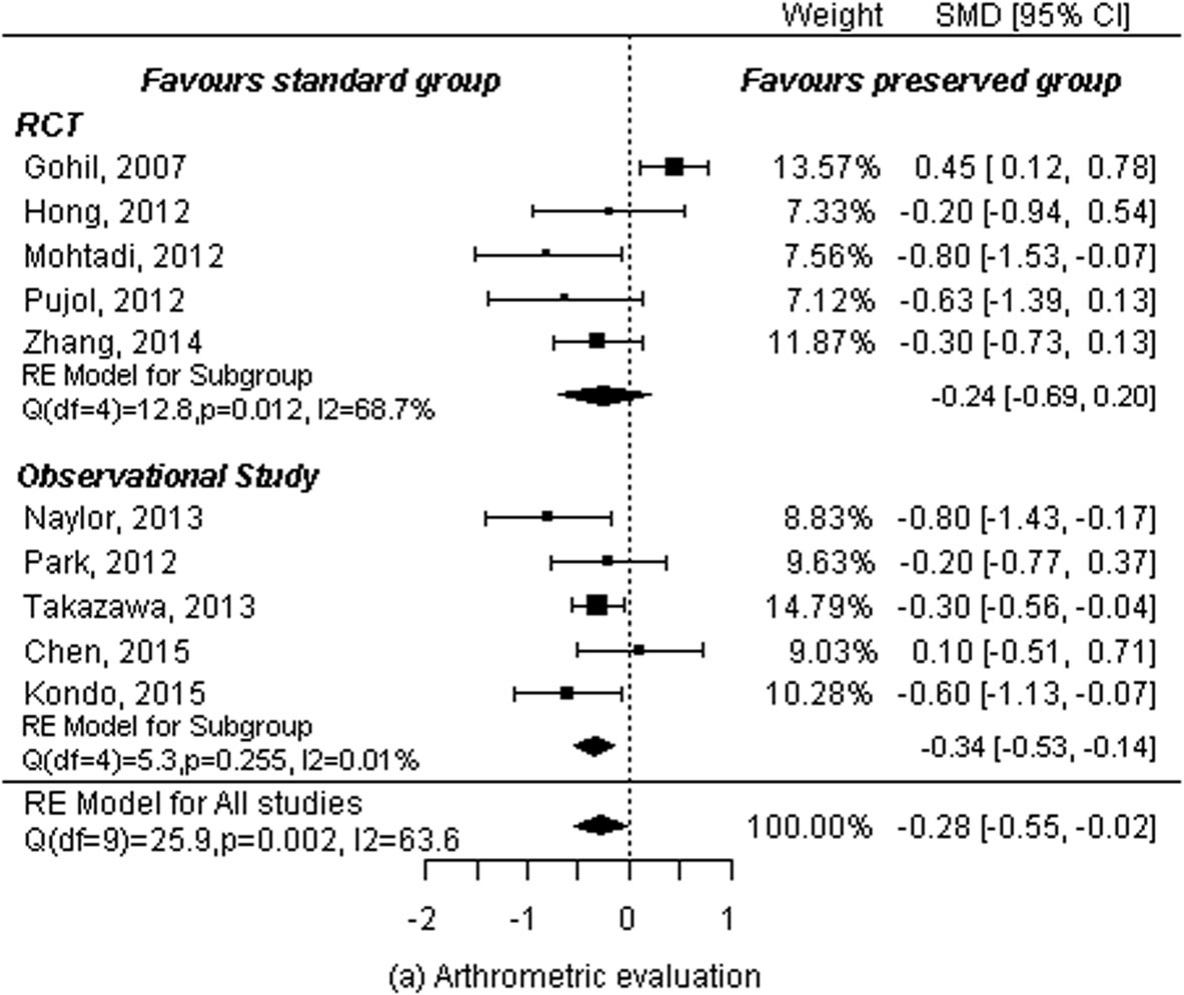
Forest plot of meta-analysis: Arthrometric evaluation

**Fig. 4:**
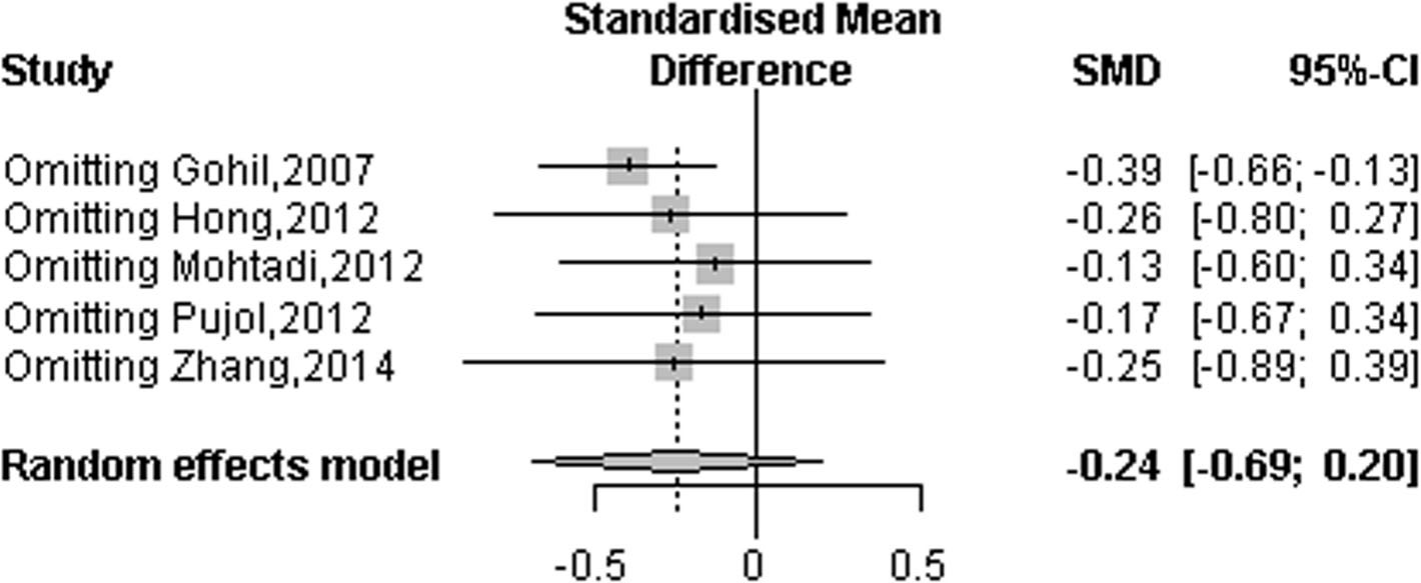
Sensitivity analysis of arthrometric evaluation

Five RCTs [5, 10–13] and five observational studies [15–19] reported arthrometric evaluation (KT-1000, KT-2000, Rolimeter, magnitude of side-to-side difference). A total of 472 patients in remnant-preserving groups and 485 patients in non-preserving groups were analyzed. A random-effects model was used to calculate summary statistics and standard mean differences to estimate overall effect sizes because different authors used different measurement units for each study. Results showed significant differences between remnant-preserving and remnant non-preserving groups across studies (SMD: -0.28, 95% CI: − 0.55 to − 0.02, *P* = 0.038). However, there was significant heterogeneity (*P* = 0.002, I^2^ = 63.66%). When subgroup analysis was performed by study design, effect magnitudes and statistical significance changed in observational studies (SMD: -0.34, 95% CI: − 0.53 to − 0.14, *P* = 0.001). There was no heterogeneity (*P* = 0.26 and I^2^ = 0.01%; Fig. 3). In contrast, there was still significant heterogeneity between studies (*P* = 0.01, I^2^ = 68.69%) among RCTs, although results of the pooled estimations were not statistically significant (SMD: -0.24, 95% CI: − 0.69 to 0.20, *P* = 0.28; Fig. 4). To address the heterogeneity that remained among the RCT subgroup, a sensitivity analysis was conducted by year. The study by Gohil et al. [10] was regarded as an outlier study because the calculated summary statistics (SMD: -0.39, 95% CI: − 0.66 to − 0.13, *P* = 0.003) showed a big difference except for the effect of the study. The heterogeneity disappeared to 0% of I^2^ after excluding that study (Fig. 4).

### Pivot shift test

**Fig. 5:**
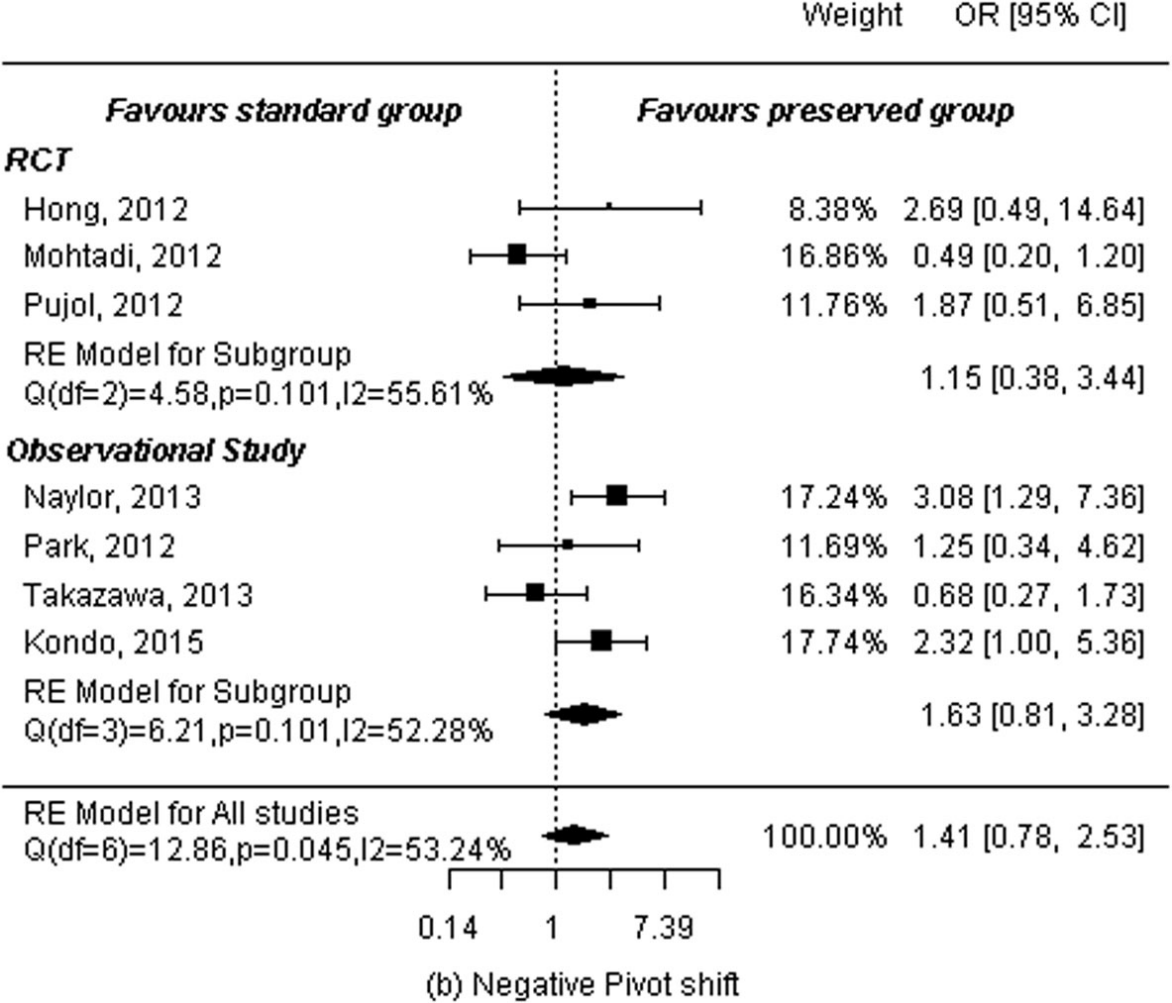
Forest plot of meta-analysis: Negative pivot shift

Grade 0 as was defined as negative pivot shift. Otherwise, it was defined as positive pivot shift. Pivot shift tests were conducted with three RCTs [11–13] and four observational studies [15–17, 19], analyzing a total of 383 patients in the remnant-preserving group and 399 in the remnant non-preserving group. A random-effects model was used to calculate summary statistics. No significant difference was found between the two groups (OR: 1.41, 95% CI: 0.78 to 2.53, *P* = 0.25). There was a low statistical heterogeneity among studies (*P* = 0.05, I^2^ = 53.24%). Subgroup analyses were then performed by study design. Analysis of three RCTs showed no significant differences between the two groups (OR: 1.15, 95% CI: 0.38 to 3.44, *P* = 0.80). There was a low statistical heterogeneity among studies (*P* = 0.10, I^2^ = 55.61%). Analysis of four observational studies showed the same results: no significant difference between the two groups (OR: 1.63, 95% CI: 0.81 to 3.28, *P* = 0.17) and low statistical heterogeneity among studies (P = 0.10, I^2^ = 52.28%; Fig. 5).

### Lachman test

**Fig. 6:**
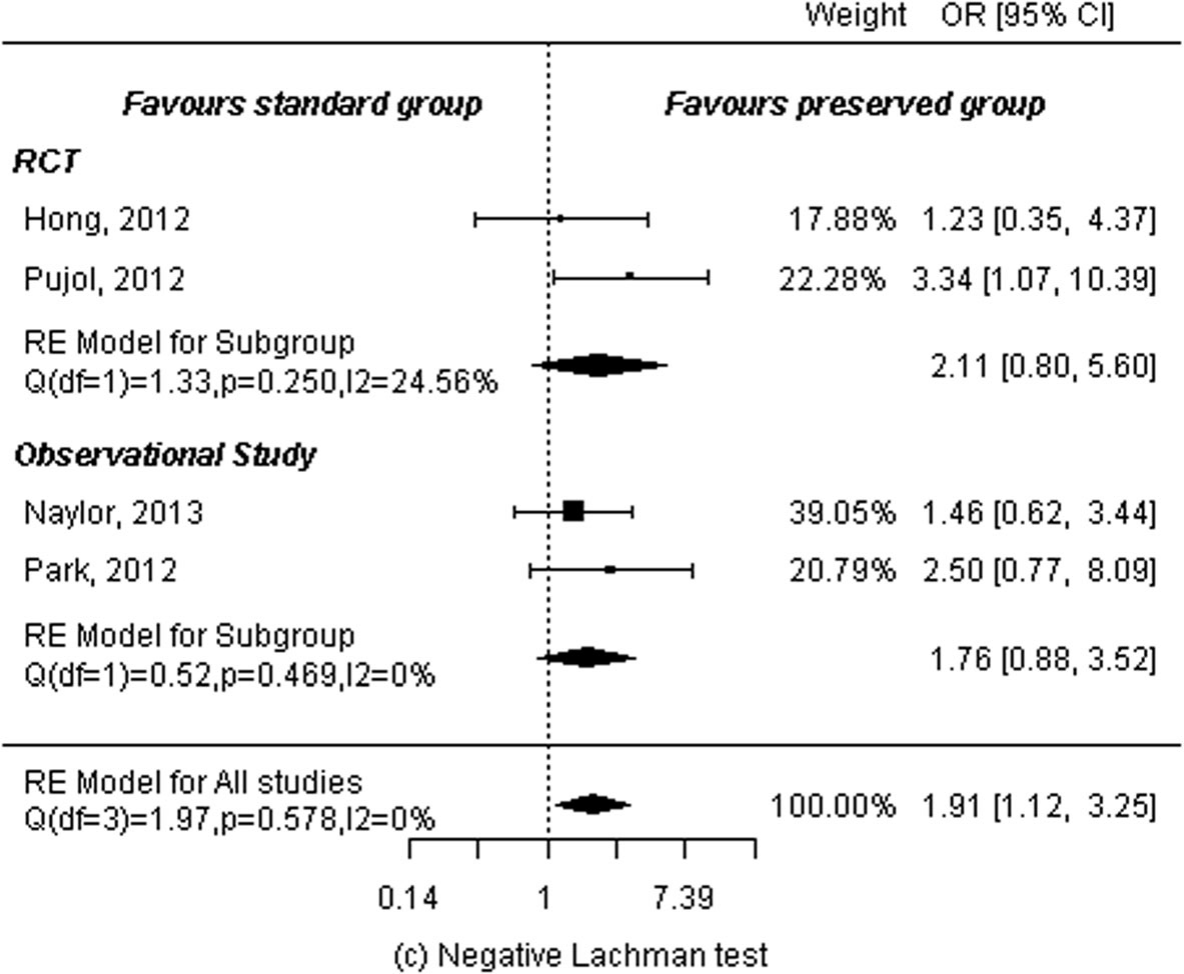
Forest plot of meta-analysis: Negative Lachmann test

Two RCTs [11, 13] and two observational studies [15, 16] reported Lachman test results. A total of 174 patients in the remnant-preserving group and 160 patients in the remnant non-preserving group were analyzed. A random-effects model was used to calculate summary statistics. Results showed significant differences between the two groups (OR: 1.91, 95% CI: 1.12 to 3.25, *P* = 0.02) and no heterogeneity among studies (*P* = 0.58, I^2^ = 0.00%). Subgroup analyses were then performed by study design. Analysis of two RCTs showed no significant differences between the two groups (OR: 2.11, 95% CI: 0.80 to 5.60, *P* = 0.13) and low statistical heterogeneity among studies (*P* = 0.25, I^2^ = 24.56%). Analysis of two observational studies showed the same results: no significant differences between the two groups (OR: 1.76, 95% CI: 0.88 to 3.52, *P* = 0.11) and no heterogeneity among studies (*P* = 0.47, I^2^ = 0.00%; Fig. 6).

### Lysholm scores

**Fig. 7:**
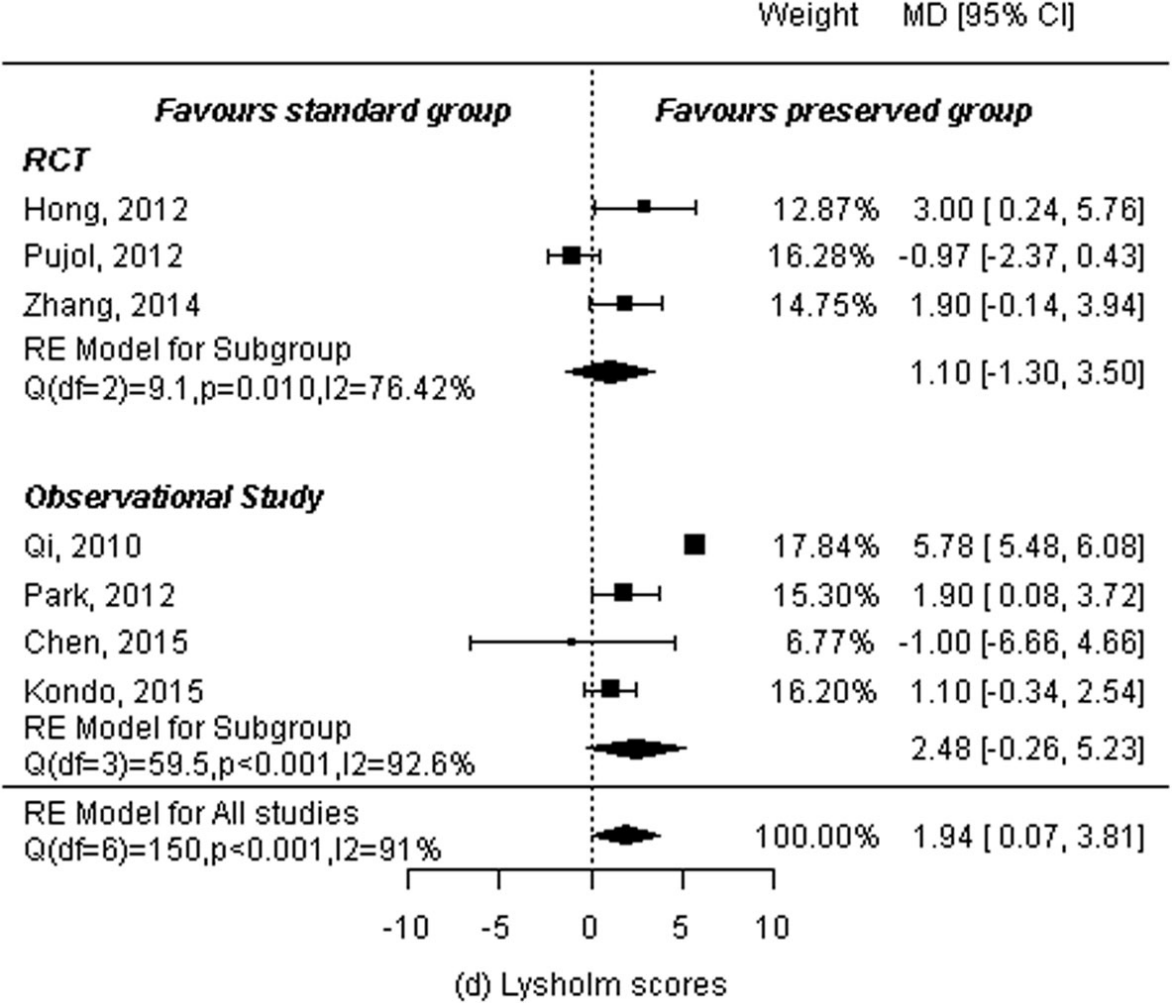
Forest plot of meta-analysis: Lysholm scores

Three RCTs [5, 11, 13] and four observational studies [14, 15, 18, 19] reported Lysholm knee scoring scale scores for a total of 312 patients in the remnant-preserving group and 333 in the remnant non-preserving group. A random-effects model was used to calculate summary statistics. Results showed statistically significant differences between the two groups (MD: 1.94, 95% CI: 0.07 to 3.81, *P* = 0.042) and statistical heterogeneity among studies (*P* < 0.001, I^2^ = 91.01%). Subgroup analyses were performed by study design. Analysis of three RCTs showed no significant differences between the two groups (MD: 1.10, 95% CI: − 1.30 to 3.50, *P* = 0.37) and significant heterogeneity (*P* = 0.01, I^2^ = 76.42%). Analysis of four observational studies showed the same results: no significant differences between the two groups (MD: 2.48, 95% CI: − 0.26 to 5.23, *P* = 0.08) and significant heterogeneity (P < 0.001, I^2^ = 92.63%; Fig. 7).

### IKDC grade

**Fig. 8:**
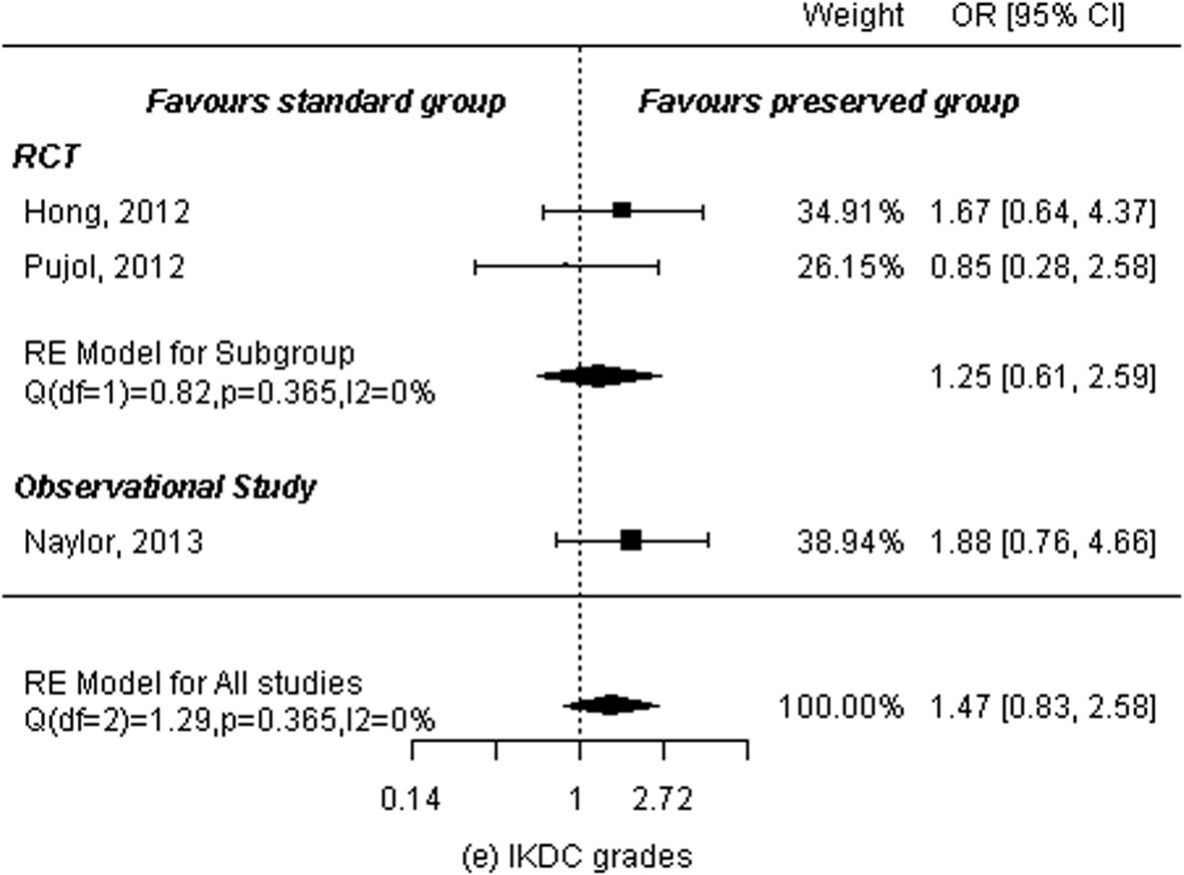
Forest plot of meta-analysis: IKDC grades

Two RCTs [11, 13] and one observational study [16] included IKDC grades for a total of 119 patients in the remnant-preserving group and 115 in the remnant non-preserving group. A random-effects model was used to calculate summary statistics. Results showed no significant difference between the two groups (OR: 1.47, 95% CI: 0.83 to 2.58, *P* = 0.19) and no heterogeneity among studies (*P* = 0.53, I^2^ = 0.00%). Subgroup analysis was performed according to study design. Analysis of two RCTs showed no significant differences between the two groups (OR: 1.25, 95% CI: 0.61 to 2.59, *P* = 0.54) and no heterogeneity among studies (P = 0.37, I^2^ = 0.00%; Fig. 8).

### IKDC subjective scores

**Fig. 9:**
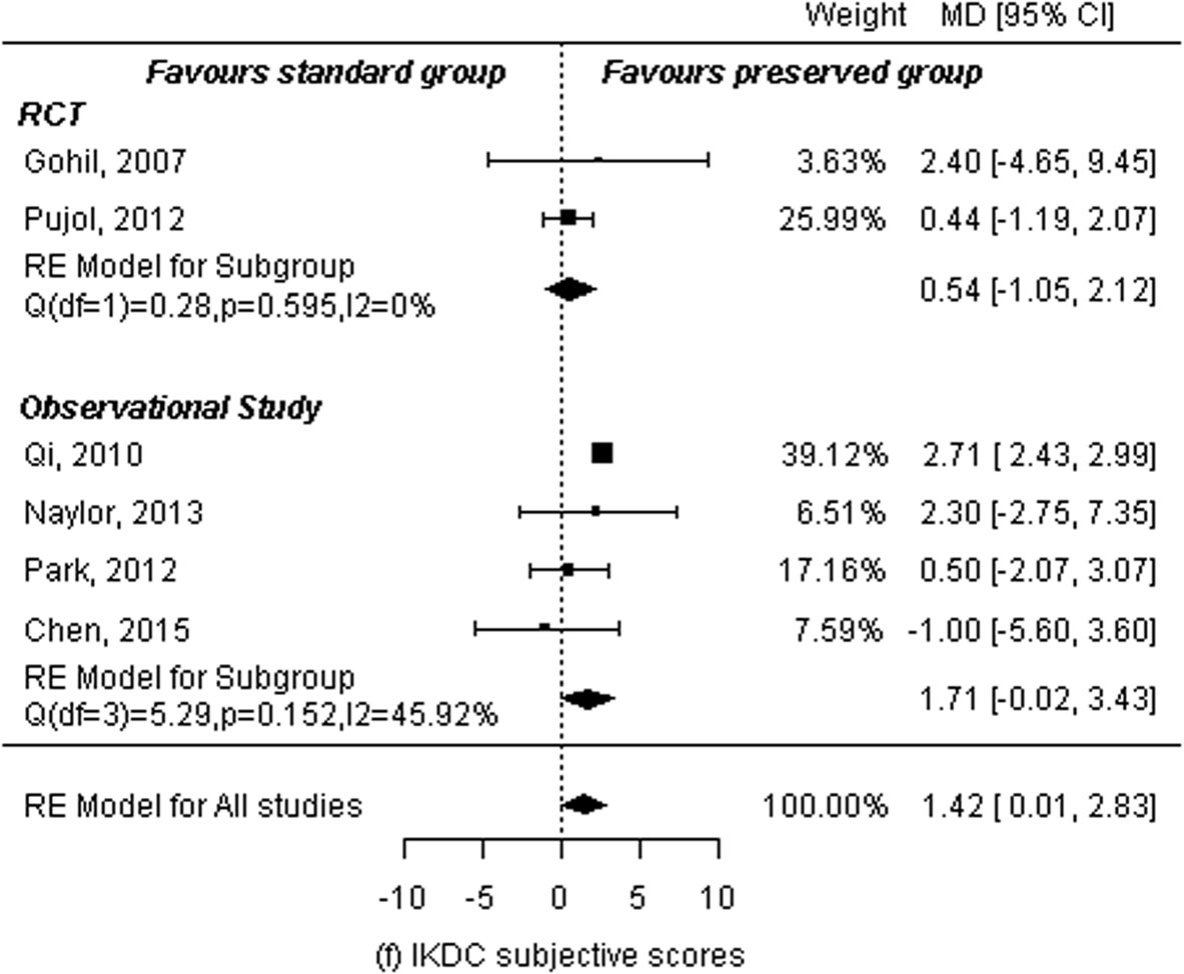
Forest plot of meta-analysis: IKDC subjective scores

Two RCTs [10, 13] and four observational studies [14–16, 18] reported IKDC subjective scores for a total of 228 patients in the remnant-preserving group and 236 in the remnant non-preserving group. A random-effects model was used to calculate summary statistics. There was statistically significant difference between the two groups (MD: 1.42, 95% CI: 0.01 to 2.83, *P* = 0.048). There was a low statistical heterogeneity among studies (*P* = 0.03, I^2^ = 55.22%). Subgroup analyses were performed by study design. Analysis of two RCTs showed no significant differences between the two groups (MD: 0.54, 95% CI: − 1.05 to 2.12, *P* = 0.50). The heterogeneity was resolved (*P* = 0.60, I^2^ = 0.00%). Analysis of four observational studies showed the same results: no significant differences between the two groups (MD: 1.71, 95% CI: − 0.02 to 3.43, *P* = 0.053) and low statistical heterogeneity among studies (*P* = 0.15, I^2^ = 45.92%; Fig. 9).

### Cyclops lesion

**Fig. 10:**
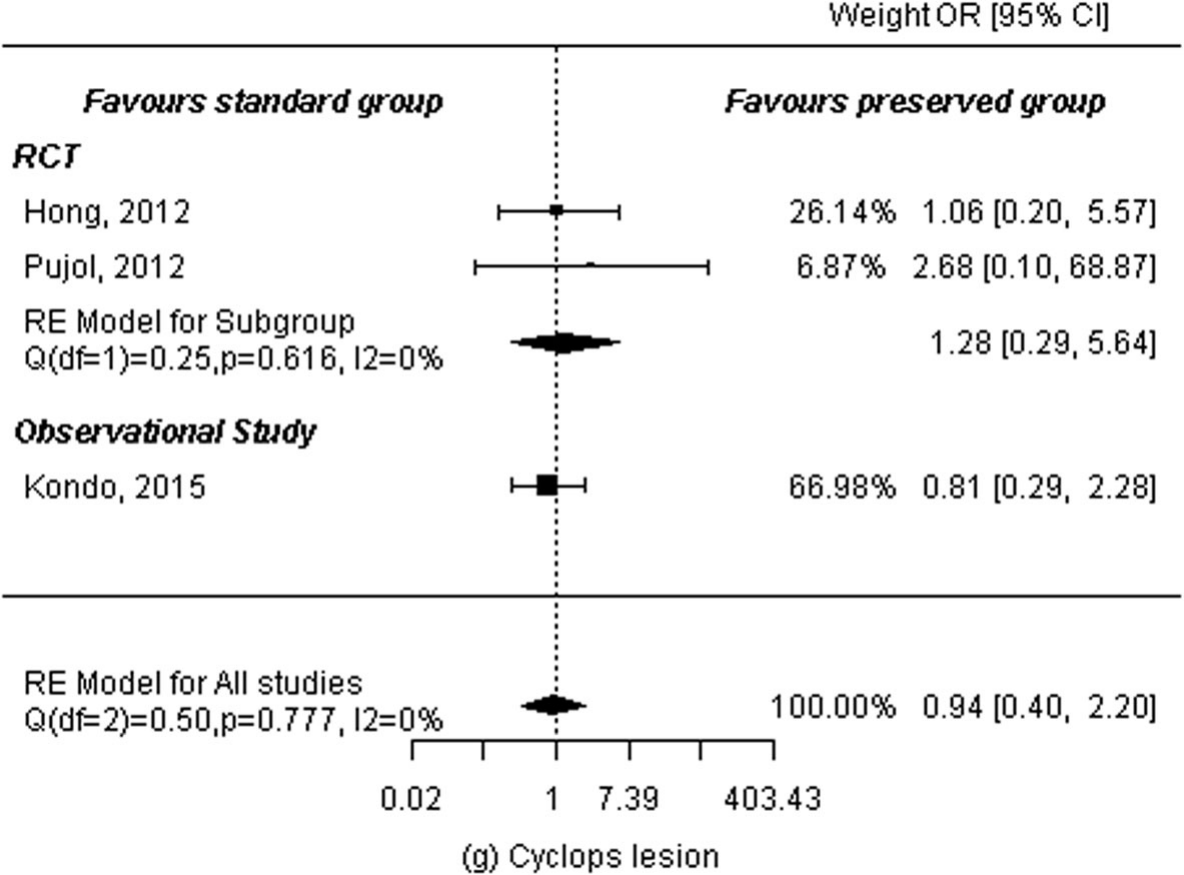
Forest plot of meta-analysis: Cyclops lesion

Two RCTs [11, 13] and one observational study [19] reported cyclops lesion among a total of 155 patients in the remnant-preserving group and 168 in the remnant non-preserving group. A random-effects model was used to calculate summary statistics. There was no significant difference between the two groups (OR: 0.94, 95% CI: 0.40 to 2.20, *P* = 0.89). There was no heterogeneity among studies (*P* = 0.78, I^2^ = 0.00%). Subgroup analyses were performed by study design. Analysis of three RCTs showed no significant difference between the two groups (OR: 1.28, 95% CI: 0.29 to 5.64, *P* = 0.74). There was no heterogeneity among the studies either (*P* = 0.62, I^2^ = 0.00). Additionally, one observational study showed no significant difference between the two groups (MD: 0.81, 95% CI: 0.29 to 2.28, *P* = 0.69; Fig. 10).

## Discussion

Investigation to reveal the value of the remnant preservation in ACL reconstruction is important in the field of sports medicine. Reflecting this, systematic review [6, 7] and meta-analysis [8, 9] on this topic have been published over the past years. The present meta-analysis investigated clinical outcome differences including mechanical stability using arthrometric evaluation, functional evaluation, and complications between remnant preserving and non-preserving method for primary arthroscopic ACL reconstruction.

The key finding of the current meta-analysis that included five RCTs and six observational studies was that the remnant-preserving method showed a statistically significant difference compared to the non-preserving method with respect to arthrometric evaluation (side-to-side difference). Results of subgroup analyses also demonstrated statistically significant difference between the two groups without heterogeneity. Lachman test, Lysholm scores, and IKDC subjective scores showed statistical difference in meta-analysis, but showed no statistical difference in subgroup analysis in each outcome parameter. Remaining outcome parameters including pivot shift test, IKDC grades, incidence of cyclops lesion showed no statistically differences in meta-analysis or subgroup analysis.

One of the goals of ACL reconstruction is to restore biomechanically stable joint. From this viewpoint, many investigators have made efforts to improve clinical results. In this respect, remnant preserving method is one of considerable efforts for primary ACL reconstruction.

After the report of arthroscopic remnant-preserving ACL reconstruction by Lee et al. [4], many studies have reported remnant-preserving techniques and their clinical outcomes. Lee et al. [20] and Ahn et al. [21] have reported good clinical outcomes after remnant-preserving ACL reconstruction. Some clinical studies [22, 23] have revealed that the remnant preserving method in ACL reconstruction can influence knee joint stability. Kondo et al. [19] have reported that the remnant-preserving method shows significantly better outcome in terms of mechanical stability and arthroscopic evaluation than the non-preserving method after anatomic double-bundle arthroscopic ACL reconstruction. Kitamura et al. [24] have evaluated intraoperative 3-dimensional kinematics using an electromagnetic sensor system and demonstrated that the remnant-preserving anatomic double-bundle ACL reconstruction appears to improve the control of pivot-shift laxity at a minimum of 12 months after surgery. Kim et al. [25] have reported that the mean postoperative arthrometric evaluation (side-to-side difference) is 1.67 mm on KT − 2000 for those who have undergone remnant-preserving double bundle ACL reconstruction. Adachi et al. [26] have reported that the mean postoperative arthrometric evaluation (side-to-side difference) in the remnant preserving group is 0.7 mm versus 1.8 mm in the non-preserving group (*P* < 0.05). In this meta-analysis, statistical results of arthrometric evaluation (side-to-side difference) were comparable to those of previous studies. Based on such results, the remnant-preserving method in ACL reconstruction may preserve a portion of blood vessels from the tibial attachment site which may accelerate revascularization of the graft, resulting in early restoration of mechanical properties of the graft. This might be one of possible reasons for better stability. There is a view that preserving remnant tissue may lead to complications such as cyclops lesion [27], also known as localized anterior arthrofibrosis with extension limitation. The incidence of a cyclops lesion related to ACL reconstruction has been reported to range from 2 to 47% [21, 28]. Many studies have suggested that remnant-preserving method does not increase the incidence of cyclops lesion [10, 11, 13, 19]. Results of our meta-analysis showed no statistically significant difference in the incidence of cyclops lesion between remnant-preserving method and non-preserving method (*P* > 0.05).

However, actual effectiveness of remnant-preserving method is still inconsistent. It is currently unclear whether there is a definitive clinical relevance or advantage over non-preserving method during arthroscopic ACL reconstruction. Although this meta-analysis results showed statistically significant difference in terms of arthrometric evaluation between the two groups, interpretation of this result should have limited clinical relevance because magnitude of the differences was not large enough (0.1–1.4 mm), the arthrometer measures anterior knee laxity in 1 mm increments of precision and IKDC considers a side-to-side difference as normal value up to 2 mm [29, 30].

Results of Lachman test, Lysholm scores, and IKDC subjective scores also demonstrated statistically minor differences. However, these minor differences did not reflect the clinical relevance. They were not large enough to distinguish clinical differences. Statistically, the calculated summary statistics, including each subgroup and all results of the study, showed different results. In addition, the 95% CI of overall summary statistics showed a slight deviation from the boundary. Point estimates were statistically significant. Therefore, a pattern of increasing statistical significance may show by increasing the number of samples. Thus, more research is needed than to conclude that it is significant.

A number of studies [11, 31] have compared clinical outcomes of remnant-preserving and non-preserving methods and found no statistically significant differences between the two groups. Ma et al. [8] have found significant differences in favor of remnant-preserving method for Lysholm scores, arthrometer measurements, and tibial tunnel enlargements. They reported no significant differences between remnant-preserving and non-preserving methods with respect to IKDC grades and scores, Lachman test results, pivot shift test results, range of motion, and incidence of cyclops lesion. Tie et al. [9] have performed a meta-analysis of RCTs and found no significant difference between groups for KT arthrometer, negative Lachman test scores, or pivot shift test results. By functional outcomes, the authors found no significant differences in IKDC scores/grades or Lysholm scores. The percentage of tibial tunnel enlargement in the remnant-preserving group was significantly lower, although there was no significant difference in the incidence of cyclops lesion. However, their meta-analysis had the following errors. The mixture of ACL partial tears RCTs with remnant- preserving RCTs (remnant-preserving vs. non-preserving ACL reconstruction) compared two different situations which could cause significant bias. Therefore, we considered ACL partial tear study should be excluded. Furthermore, randomized controlled trials in this meta-analysis have unclear quality of remnant. They have a risk of bias because the method of blinding is not reported.

Our meta-analysis and subgroup analysis have some limitations that should be taken into account. First, although we assessed the quality of included observational studies using quality assessment tool (NOS) and found that all of them had good quality, observational study itself has a lower level of evidence than RCT. Second, there was heterogeneity between studies with regard to patient characteristics, graft types, and surgical techniques, all of which might have affected outcomes. Third, clinical outcomes used in studies could not directly support the role of remnant-preserving method in ACL reconstruction. Fourth, despite statistically significant differences in terms of arthrometric evaluation, Lachman test, Lysholm scores, and IKDC subjective scores, the magnitude of difference was not large enough. Therefore, it is difficult to interpret that remnant-preserving arthroscopic ACL reconstruction method provides superior outcomes than non-preserving method. Thus, precautions are required when interpreting these results. Fifth, both RCTs and observational studies have only about 1 year of follow-up duration in seven of eleven studies. The outcome might be different with longer follow-up duration.

## Conclusion

This meta-analysis with subgroup analysis showed that arthroscopic remnant-preserving ACL reconstruction provided statistically significant but limited clinical relevance in terms of arthrometric evaluation. Results of Lachman test, Lysholm scores, and IKDC subjective scores demonstrated statistically minor differences.

## Data Availability

All data generated or analyzed during this study are included in this published data.

## Abbreviations

ACL: Anterior cruciate ligament
CI: Confidence intervals
ICC: Intraclass correlation coefficient
IKDC: International Knee Documentation Committee
NOS: Newcastle-Ottawa Scale
OR: Odd ratio
PEDro: Physiotherapy Evidence Database
PRISMA: Preferred Reporting Items for Systematic Review and Meta-Analysis
RCTs: Randomized controlled trials
RR: Relative risk
SIR: Standardized incidence ration
SMD: Standardized mean difference
